# Multispectral tissue mapping: developing a concept for the optical evaluation of liver disease

**DOI:** 10.1117/1.JMI.7.6.066001

**Published:** 2020-12-23

**Authors:** Crispin Schneider, Daniil Nikitichev, Wenfeng Xia, Kurinchi Gurusamy, Adrien E. Desjardins, Brian R. Davidson

**Affiliations:** a University College London, Division of Surgery and Interventional Science, Royal Free Campus, London, United Kingdom; b University College London, Wellcome/EPSRC Centre for Surgical and Interventional Sciences, London, United Kingdom; c University College London, Department of Medical Physics and Bioengineering, London, United Kingdom

**Keywords:** multispectral imaging, hyperspectral imaging, liver cancer, liver steatosis, false color imaging

## Abstract

**Purpose:** Alterations in the optical absorption behavior of liver tissue secondary to pathological processes can be evaluated by multispectral analysis, which is increasingly being explored as an imaging adjunct for use in liver surgery. Current methods are either invasive or have a limited wavelength spectrum, which restricts utility. This proof of concept study describes the development of a multispectral imaging (MSI) method called multispectral tissue mapping (MTM) that addresses these issues.

**Approach:** The imaging system consists of a tunable excitation light source and a near-infrared camera. Following the development stage, proof of concept experiments are carried out where absorption spectra from colorectal cancer liver metastasis (CRLM), hepatocellular carcinoma (HCC), and liver steatosis specimen are acquired and compared to controls. Absorption spectra are compared to histopathology examination as the current gold standard for tissue assessment. Generalized linear mixed modeling is employed to compare absorption characteristics of individual pixels and to select wavelengths for false color image processing with the aim of visually enhancing cancer tissue.

**Results:** Analysis of individual pixels revealed distinct absorption spectra therefore suggesting that MTM is possible. A prominent absorption peak at 1210 nm was found in lipid-rich animal tissues and steatotic liver specimen. Liver cancer tissue had a heterogeneous appearance on MSI. Subsequent statistical analysis suggests that measuring changes in absorption behavior may be a feasible method to estimate the pixel-based probability of cancer being present. In CRLM, this was observed throughout 1100 to 1700 nm, whereas in HCC it was concentrated around 1140 and 1430 nm. False color image processing visibly enhances contrast between cancer and normal liver tissues.

**Conclusions:** The system’s ability to enable no-touch MSI at 1100 to 1700 nm was demonstrated. Preliminary data suggest that MTM warrants further exploration as a potential imaging tool for the detection of liver cancer during surgery.

## 1 Introduction

Pathological processes can alter the light absorption of tissues within the visible and invisible spectrum of light. Specific absorption characteristics can be elucidated by plotting tissue absorption at different light wavelengths. These characteristics are based on substances (e.g., lipids or water) within tissues that have one or more absorption maxima (a wavelength where more light is absorbed than in the adjacent wavelengths) that is specific to them and that can be used to infer their respective presence and in some cases to estimate their tissue concentration.^1^ This principle can also be employed to visualize anatomical details that are not apparent to the naked eye (e.g., blood vessels within mesentery).^2^

In liver disease, it has been demonstrated that probe-based diffuse optical spectroscopy may reveal changes in tissue composition that are indicative of pathological processes such as malignancy or steatosis.^3^^,^^4^ Because this method allows discrimination between diseased and healthy tissues, it can be regarded as a potential diagnostic or prognostic test. Utilizing diffuse optical spectroscopy in an experimental study of hepatocellular cancer (HCC), a reduction in lipid, hemoglobin, and tissue oxygenation was found to be associated with HCC.^5^ In analogy to these results, a study on resected human liver specimens containing colorectal cancer liver metastases (CRLM) showed altered absorption spectra in cancerous tissue secondary to a reduction in lipid, bile, and hemoglobin content.^4^

On this basis, a technology that can distinguish between cancerous and healthy tissues would have wide applications in healthcare as a diagnostic tool. Applied to liver surgery, it could enable surgeons to confirm the complete resection of a tumor. Furthermore, the objective, intraoperative estimation of liver steatosis could enhance the qualitative assessment of the functional liver remnant, which is an important step in avoiding small-for-size syndrome after major hepatectomies.^6^

Although the results from the aforementioned studies are promising, there are several disadvantages to employing a probe-based design intraoperatively. First, the tissue evaluation is limited to a small volume of tissue due to the small separation distance between the optical fibers delivering and receiving light (1.84 mm). This means it may take a long time to assess a clinically meaningful area. Second, the tissue analysis depends on changes in the spectroscopic response curve, which is not an intuitive or time efficient way to mentally process information during surgery. Finally, a probe needs to be in direct contact with tissue that makes it less suitable for minimal invasive surgery where access and maneuverability may be limited.

An alternative to probe-based optical spectroscopy is to utilize a visual approach where spectral data can be inferred from images (e.g., video or photo). With this approach, changes in absorption are translated into pixel intensities (pixel brightness). This method is sometimes referred to as multispectral or hyperspectral imaging because tissues are viewed at multiple different wavelengths.^7^

The feasibility of multispectral imaging (MSI) of the liver has previously been demonstrated by different authors that utilized a laparoscope fitted with a light filter that narrows the reflected (i.e., received by the laparoscope) light down to a specific spectral range (e.g., range of 20 nm). Selective filtering is controlled by a liquid crystal tunable filter (LCTF)^8^ or through utilization of a multiple bandpass filter.^2^ This method enabled laparoscopic evaluation of relative hemoglobin concentration,^8^ which improved visualization of superficial blood vessels.^2^ LCTF focusing on determination of fat, water, and hemoglobin contents in a porcine model has been shown to discriminate between different anatomical structures (hepatic artery, portal vein, and the common bile duct) entering the liver within the hepatoduodenal ligament.^9^ Although MSI in this form enables a no-touch evaluation of liver tissue and is hence advantageous for intraoperative use, the devices to date have been limited to wavelength ranges of 420 to 700 nm^7^ or 650 to 1100 nm.^9^ This range is unlikely to enable a detailed tissue discrimination as has been reported for the probe-based diffuse reflectance spectroscopy.^4^

The aim of this study is to describe the development and proof of concept experiments of a new MSI technique that combines the advantages of both techniques, namely remote noncontact evaluation of liver tissue with a broad wavelength range of >500  nm. The proposed technique termed multispectral tissue mapping (MTM) uses a tunable excitation laser in combination with a near-infrared (NIR) camera to create detailed spectral response curves.

## 2 Materials and Methods

### 2.1 General Setup

In this study, the spectroscopic response curves are recorded from liver tissue samples. This is achieved by illuminating tissue with an optical parametric oscillator, a light source that can be tuned to wavelengths between 700 and 2000 nm. A NIR spectrum camera with an indium–gallium–arsenide sensor (InGaAs) is employed to record the reflected light, which is measured as a pixel intensity on an arbitrary scale of 0-16.383.

Light absorbed by tissues is inversely related to the amount of reflected light. Therefore, higher pixel intensities (=higher reflection) translate to higher tissue scattering and lower tissue absorption. By changing the excitation wavelength over time, it is possible to record image-based absorption curves (spectra) that are spatially correlated with the examined liver tissue. At the conclusion of experiments, the specimen biopsies undergo histological examination to confirm pathological changes.

### 2.2 *Ex Vivo* Tissues for Multispectral Evaluation

During the initial development of the imaging platform, animal tissue intended for consumption was used in experiments due to its wide availability. When the development phase was concluded, the experiments continued with liver tissue excised from patients undergoing liver resection and from discarded donor livers [i.e., livers that were initially intended for organ donation but that were found to be unsuitable due to increased fat content (liver steatosis)]. Pathological samples were compared to normal liver tissue either from the same patient in liver cancer or a different patient in liver steatosis. Due to the early development stage of the system, the experiments were conducted as a proof of concept and therefore no formal power calculation was carried out. The aim was to assess the imaging methodology on two types of liver malignancy and on liver steatosis. The experiments were not intended to proof a causal relationship between liver pathology and absorption characteristics.

### 2.3 Patient Tissue

Ethical approval for the research use of patient tissues obtained following informed consent for research at the Royal Free Hospital, London, United Kingdom, was granted by the UCL Biobank Ethical Review Committee (Reference Number NC2015.012). All experiments in this paper were performed in accordance with the UCL Biobank guidelines and regulations. Tissue from patients undergoing liver resection for CRLM or HCC was collected after surgery. The size of the specimen was tailored to fit under microscope glass slides (75×25  mm). Frequently, however, the maximal size was limited by the available liver tissue especially in small volume liver resections. The minimum requirement for tissue collection in cancer patients was to obtain at least one sample of healthy and cancerous tissues for each patient. Out of eight patients, three were excluded from analysis, because it was not possible to excise sufficient tissue without compromising histopathological analysis.

Specimen were transported in cooled (∼4°C) organ storage solution (KPS-1™, Organ Recovery Systems, Itasca, Illinois). During imaging, specimens were placed on a translation stage that enabled fine adjustment of the distance between camera and specimen. To reduce specular reflections, the specimens were covered with microscope glass slides.

### 2.4 Configuration and Setup of the Multispectral Imaging Platform

Light excitation is conferred via an optical parametric oscillator (VersaScan L-532, GWU-Lasertechnik, Erftstadt, Germany) light source that is tunable within a wavelength range of 700 to 1000 nm and 1100 to 2000 nm. The idler beam of the OPO was coupled with an optical fiber with silica-core/silica-cladding with a core diameter of 910  μm (FG910LEC, Thorlabs, Newton City, New Jersey). Excitation occurred at a fixed repetition rate of 10 Hz and pulse duration of 10 ns. A detailed description of the system can be found here.^10^ For acquisition of multispectral images, the wavelength was altered at 5 to 10 nm intervals while NIR images were recorded with an InGaAs sensor camera (C10633-23, Hamamatsu, Hamamatsu City, Japan) with a spectral range of 950 to 1700 nm (henceforth called NIR camera). Images were saved, processed, and analyzed with a Windows™ laptop.

To avoid overillumination, additional optical filters of six optical densities were positioned between laser source and optical output fiber. Before experimental use, the OPO was calibrated to ensure an accurate wavelength output with an error margin of ∼5  nm. The validity of the calibration was checked at regular intervals with a spectrometer (NIRQuest™ series, Ocean Optics, Largo, Florida). While the laser was in operation, protective eye wear had to be worn to avoid eye injuries.

**Fig. 1:**
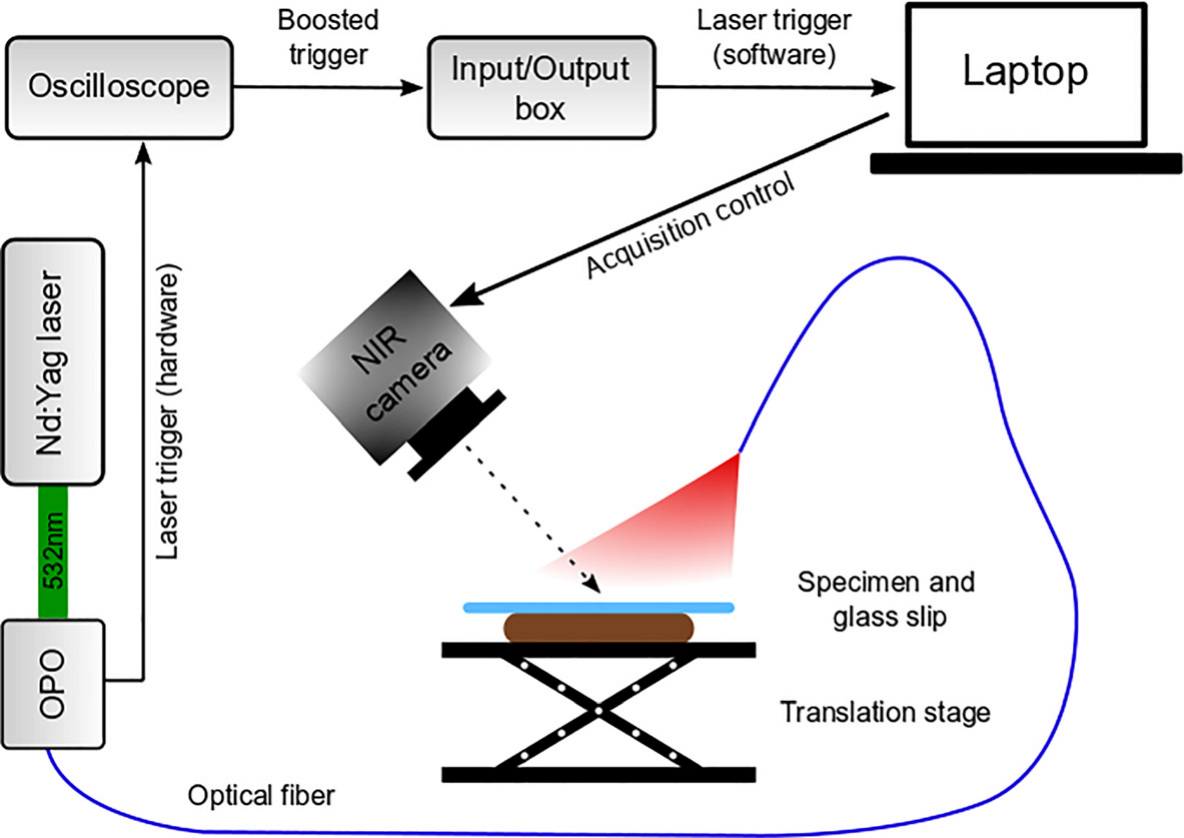
Schematic of the MTM setup.

The camera and optical fiber were positioned on posts and subsequently fixed to optical breadboards (Thorlabs). The distance between camera and specimen could be altered by raising or lowering the translation stage (Fig. 1). The NIR camera and image recording were controlled with a custom-built code written in Labview™ Professional (National Instruments, Austin, Texas).

### 2.5 Image Recording and System Synchronization

To reduce background illumination, experiments were conducted in a dark room. The lens aperture was adjusted to keep maximum pixel intensity below 16.383 (maximum limit of NIR camera). Beyond this value, over-illumination (i.e., white out) occurs, which can result in loss of data because pixel intensities above the maximum range cannot be analyzed. Prior to image acquisition, images were recorded without any illumination to determine the background intensity. To reduce the variability in illumination, pixel values were averaged over 20 frames per measured wavelength.

Discrepancies in the frame rate and exposure time between the NIR camera and the excitation light source, respectively, meant that the image acquisition was inconsistent due to a high number of lost frames. Therefore, initial development was directed at synchronizing laser excitation and camera frame rate to enable efficient image acquisition. A detailed description of the synchronization method is beyond the scope of this article. In brief, a hardware signal elicited from the OPO for every emitted pulse was amplified with the aid of an oscilloscope (Tektronix™, Beaverton, Oregon) and converted into a software signal. The latter was incorporated into the Labview image acquisition algorithm where it enabled triggering of the NIR camera. Failure to synchronize was rare (<1%) and resulted in automatic rejection of the image. Image acquisition continued until 20 synchronized images were recorded.

Spectral images were recorded at a resolution of 320×256  pixels (maximal resolution of the NIR camera) as grayscale pictures in the uint16 format. Image data were subsequently processed and analyzed with MATLAB™ (MathWorks, Natick, Massachusetts). Essentially, the gray levels between 0 and 16.383 represented all possible pixel intensities between black (=0) and white (=16.383). Therefore, high pixel intensity correlates with a low absorption and vice versa. Absorption plots can be created for a wide region of interest (ROI) or a single pixel.

### 2.6 Normalization of Absorption Spectra

For initial analysis, the spectra are obtained from wide ROIs. Each ROI corresponds to a different types of tissue. Background noise is accounted for by subtracting the average value of nonilluminated frames that were recorded prior to each experiment. Subsequently, the characteristics of the spectral curves are visually compared against each other.

To enable statistical analysis, the recorded pixel intensities were normalized to account for between experiments variation in illumination area, lens to specimen distance, light source power, specimen size, and specular artefacts. Spectra were normalized to their own maxima, which were set to a value of one. Because maxima are derived from single pixel, the absorption curves representing mean values of multiple pixels usually have a maximum below one. This approach puts more focus on the shape of the absorption curve instead of the overall illumination intensity.^11^^,^^12^

### 2.7 Statistical Analysis

**Fig. 2:**
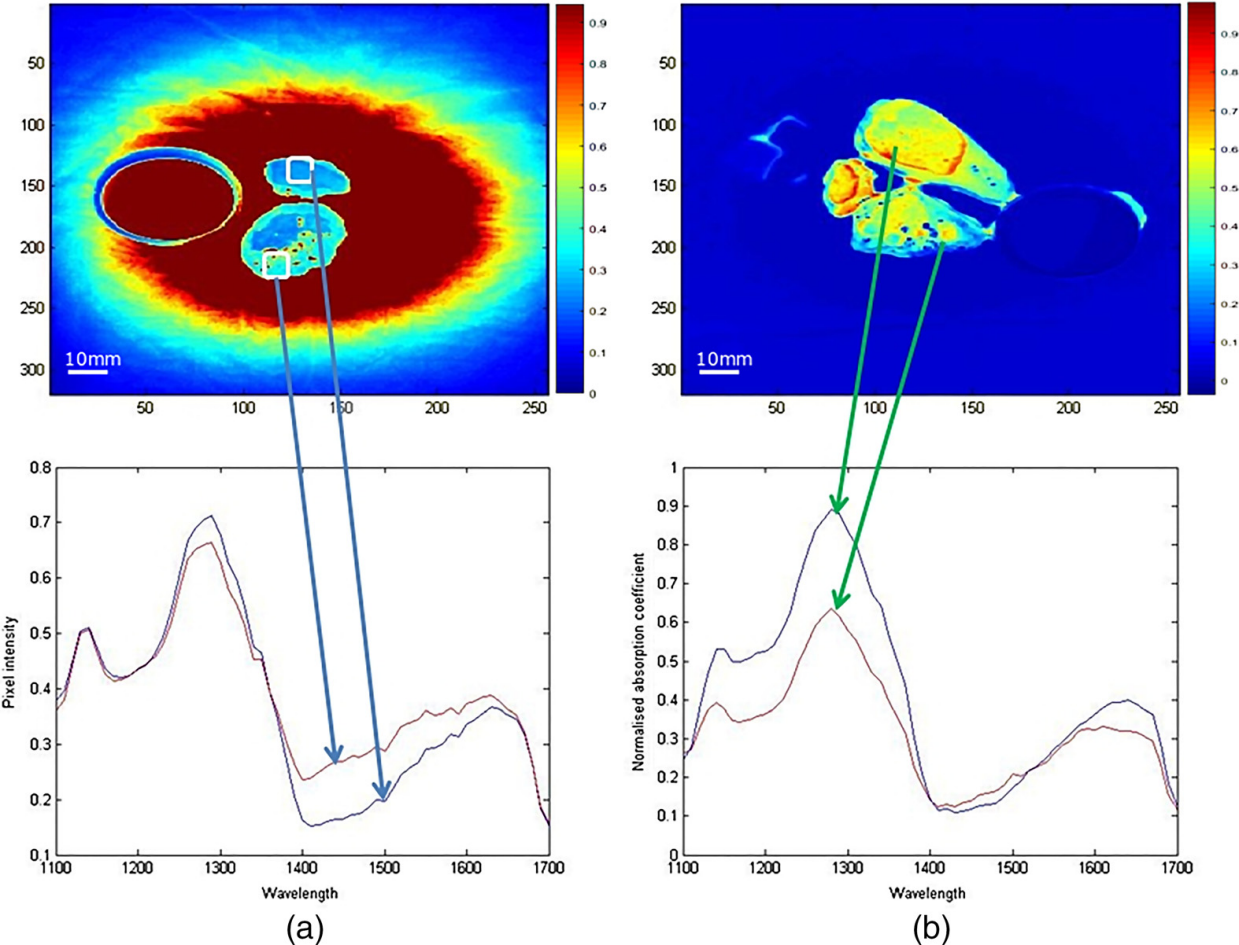
Spectral response curves can be plotted from larger tissue areas consisting of multiple pixels within an ROI. (a) Two ROIs from a patient with HCC (patient ID C01) that exhibit different absorption behaviors (arrow indicating absorption spectrum corresponding to ROI). Alternatively, a spectral response may be plotted for single pixels. (b) The arrows indicate pixels and their corresponding absorption spectra in a patient with CRLM (patient ID C03). Color bars at the side of the image indicate the relationship between measured pixel intensity and the corresponding pixel color in the image.

Following qualitative assessment of absorption spectra, different methods for the statistical analysis of multispectral data were evaluated for use in future studies of MTM. A suitable method that enables pixel rather than region of interest-based evaluation is proposed and outlined below (Fig. 2). A pixel-based assessment was felt to be optimal because the images of cancer samples appeared to be more heterogeneous (i.e., mixture of bright and dark areas) than the images from normal liver tissue. The mean value of an ROI containing several hundred pixels reflects variations in absorption behavior poorly and therefore analysis of individual pixels was felt to be preferable. Normalized pixel intensities were grouped according to tissue origin (normal tissue versus cancer), wavelengths, patient ID, and cancer type (CRLM or HCC). Underlying pathology was validated by histopathological analysis as the gold standard for tissue evaluation.

It was hypothesized that statistical data analysis using a generalized linear mixed model (GLIMMIX) could determine if variations in multispectral absorption spectra are associated with the presence of liver pathology. The advantage of GLIMMIX over conventional regression analysis is that it can analyze nonparametric, repeated measures (pixel intensities repeat for each wavelength), and that it can incorporate random and fixed variables. Fixed effects in GLIMMIX work in analogy to a regular linear or logistic regression analysis in that a covariate (e.g., age) is assigned a fixed coefficient that determines its impact on the dependent or response variable (e.g., morbidity) in the statistical model. A random effect in contrast can have different coefficients associated with a subgroup (e.g., ward in a hospital) in a dataset. The combination of fixed and random effects may allow elucidation of a better statistical model fit in some circumstances. The difference between linear mixed models and generalized linear mixed models is that the relationship between the dependent variable and the effect terms is nonlinear. This nonlinearity is expressed via a link function, which utilizes a transformation (e.g., logit, probit, log) between the effect terms and dependents in the equation. For the data in this study, for example, a binominal distribution with a logit link function was used to conduct the GLIMMIX analysis. The logit link function was chosen because it is the default GLIMMIX link function recommended for data with binominal distribution. Inclusion of random variables in the model is crucial because pixel intensities recorded on different days are not directly comparable due to differences in camera distance, sample size, lens aperture, and fluctuations in light source power. The generalized linear mixed model analysis was conducted using the Proc GLIMMIX function in SAS™ 9.4 (SAS institute, Cary, North Carolina). Normalized pixel intensity in combination with wavelengths is used as a fixed interaction term, whereas patient ID (representative of different experimental days) is used as a random term. The relationship between absorption at a given wavelength and tissue quality (i.e., normal versus cancer) is given as odds ratios (OR). OR with 95% confidence intervals (CI) are stated in relation to an increase in normalized pixel intensity of 0.25 (range 0 to 1.0), which corresponds to the interquartile range of all recorded pixel intensities.

The Proc GLIMMIX method in SAS automatically provides OR, CI, and p-values associated with covariates in the statistical model (i.e., wavelength). In brief, the GLIMMIX output provides estimates of coefficients and their corresponding covariates. The natural antilogarithm of the coefficient can subsequently be used to give an OR estimate, which in conjunction with the standard error enables calculation of CI. Finally, the p-value can be calculated utilizing log transforms of 95% CI and z-statistics.^13^ Further details on the underlying statistical methodology of GLIMMIX can be found here^14^ and in the SAS manual.

Statistical significance is not explicitly evaluated since it is a proof of concept study but tendencies can be appreciated from the OR plots below. Spectral data were analyzed individually for CRLM (n=3) and HCC (n=2). Model output includes OR, which hypothetically indicate if a change in pixel intensity at a given wavelength changes the probability of cancer tissue being present.

### 2.8 Representation of Multiple Spectra Using the False Color Method

MSI can be analyzed in a conventional way using plotted absorption spectra. Alternatively, a visual representation of spectral characteristics can be created by combining data from single or multiple wavelengths into a single composite image. In a perhaps simplified way, this could be described as “squeezing” the data contained in an absorption curve into a single image. The resulting image may be visualized in graylevels or it can be transformed into a color image. In this study, graylevels were used for visualization. The resulting composite image incorporates two-dimensional spatial data as well as multispectral data from different wavelengths. Because the representation does not correlate with real life colors or graylevels, these images are sometimes termed as false or pseudocolor images.^15^

In its simplest form (false color mean intensity), the false color representation was achieved by calculating the mean intensity across all recorded wavelengths (1100 to 1700 nm). It was hypothesized that combining different wavelength combinations may enhance the visualization and differentiation of malignant tissue. Wavelengths were chosen according to their OR on GIMMIX analysis (i.e., very high or low OR) because in theory absorption at these wavelengths should change significantly depending on the tissue type.

## 3 Results

### 3.1 Assessment of Technical Feasibility and Spatial Resolution

At first, the experiments were carried out to evaluate the optimal imaging setup and spatial resolution of MTM. These experiments were conducted without synchronization between camera and light excitation source. A low OPO power output at 900 to 1000 nm resulted in under-illumination and very dark images. It was possible to adjust this by widening the lens aperture but this unfortunately meant that subsequent images at higher wavelengths were grossly overilluminated and could therefore not be analyzed. For technical reasons, the OPO could not be tuned to wavelengths between 1000 and 1100 nm. Because of these issues, MSI acquisition was intentionally limited to wavelengths from 1100 to 1700 nm. Due to the lack of light source and NIR camera synchronization, a large number of frames were underilluminated, and hence a scatter plot representation was used to assess absorption curves during early experiments.

**Fig. 3:**
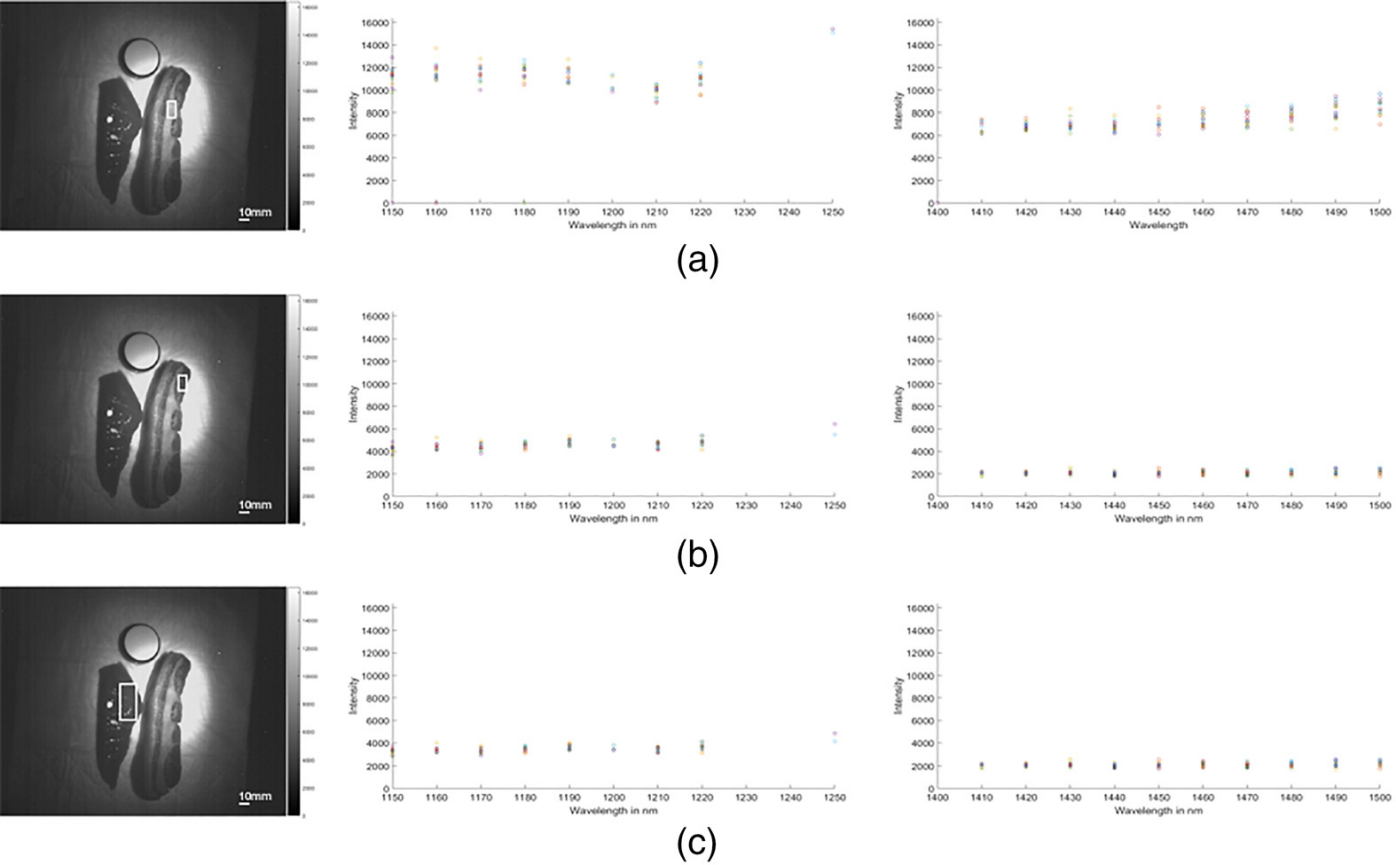
The absorption spectra for (a) bacon fat tissue, (b) bacon muscle tissue, and (c) bovine liver tissue. The figures on the left show the ROI marked with a white square. The figures in the center show spectra between 1150 and 1250 nm and the images on the right show spectra between 1400 and 1500 nm. Color bars on the side of the multispectral images are references for the relationship between grayscale shades and pixel intensity (also see in the figures below). Note that data for some wavelengths are missing due to issues with synchronization between light source and NIR camera image acquisition.

Bovine liver and streaky bacon was used for initial experiments. The bacon was thought to be useful because of its contrast between lipid-rich (fatty stripe) and water-rich (red stripe) tissues. Bovine liver was used to emulate human liver samples. MTM was repeated three times at 1150 to 1250 nm and 1400 to 1500 nm for each specimen (Fig. 3). These spectra were chosen because the former contains the lipid absorption peak around 1200 nm, and the latter contains a water absorption peak, which is broader at around 1450 nm.^16^ In all tissues, light absorption at 1400 to 1500 nm was higher than at 1150 to 1250 nm. Inspection of absorption curves revealed that spectra differed between tissues, e.g., bacon versus liver and between distinct areas in the same tissue, respectively. An absorption peak at 1210 nm, which was probably related to high lipid content of animal fat tissue, was recorded in two out of three experiments.^17^

**Fig. 4:**
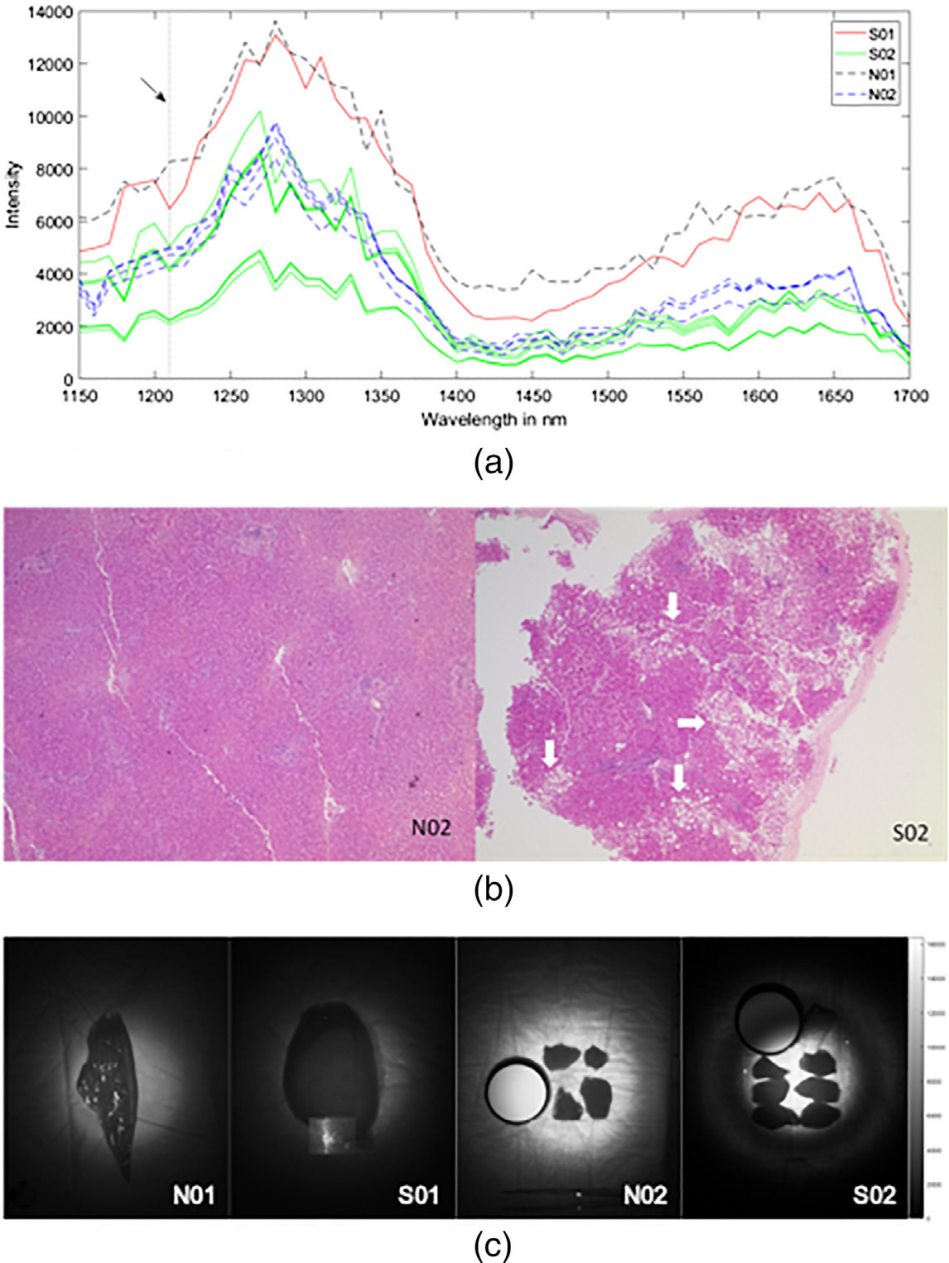
(a) Tissue spectra from ROIs of steatotic (S01 and S02: solid lines) and nonsteatotic (N01 and N02: dashed lines) liver samples from patients (n=2 each). The dotted vertical line (arrow) marks 1210 nm where an increased absorption was observed in steatotic liver tissue. Background noise has been subtracted from the nonnormalized intensity values. (b) Nonsteatotic (N02) and steatotic liver samples from patient viewed on H&E histology slides. Areas of vacuole formation (arrows) correspond with lipid deposits. (c) Multispectral images (mean intensity) of the steatotic and nonsteatotic liver specimens.

To further evaluate the impact of high lipid content on absorption characteristics, steatotic liver samples were compared to nonsteatotic samples. In analogy to the experiments on animal tissues, a prominent absorption peak at 1210 nm indicating greater lipid content in the steatotic samples was observed (Fig. 4). An increase in absorption translates to a reduction in pixel intensity (i.e., reflection) at 1210 nm as highlighted in Fig. 4(a). The presence of liver steatosis as indicated by vacuole formation was confirmed on H&E stain histology examination (Fig. 4).

Spatial resolution was extrapolated from the size of the microscope glass cover slips (75×25  mm) by measuring the number of pixels at the short and long axes of the glass slip. At a relatively constant imaging distance of 15±2  cm, the area covered by one pixel was in the range of 0.48 to $0.55\;{\mathrm{mm}}^{2}$ with an average of $0.5\;{\mathrm{mm}}^{2}$. Based on this data, and because image acquisition was improved due to the successful synchronization between laser excitation source and NIR camera, it was felt to be reasonable to expand further MSI experiments onto liver tissue from patients.

### 3.2 Analysis of Liver Cancer Specimen

**Fig. 5:**
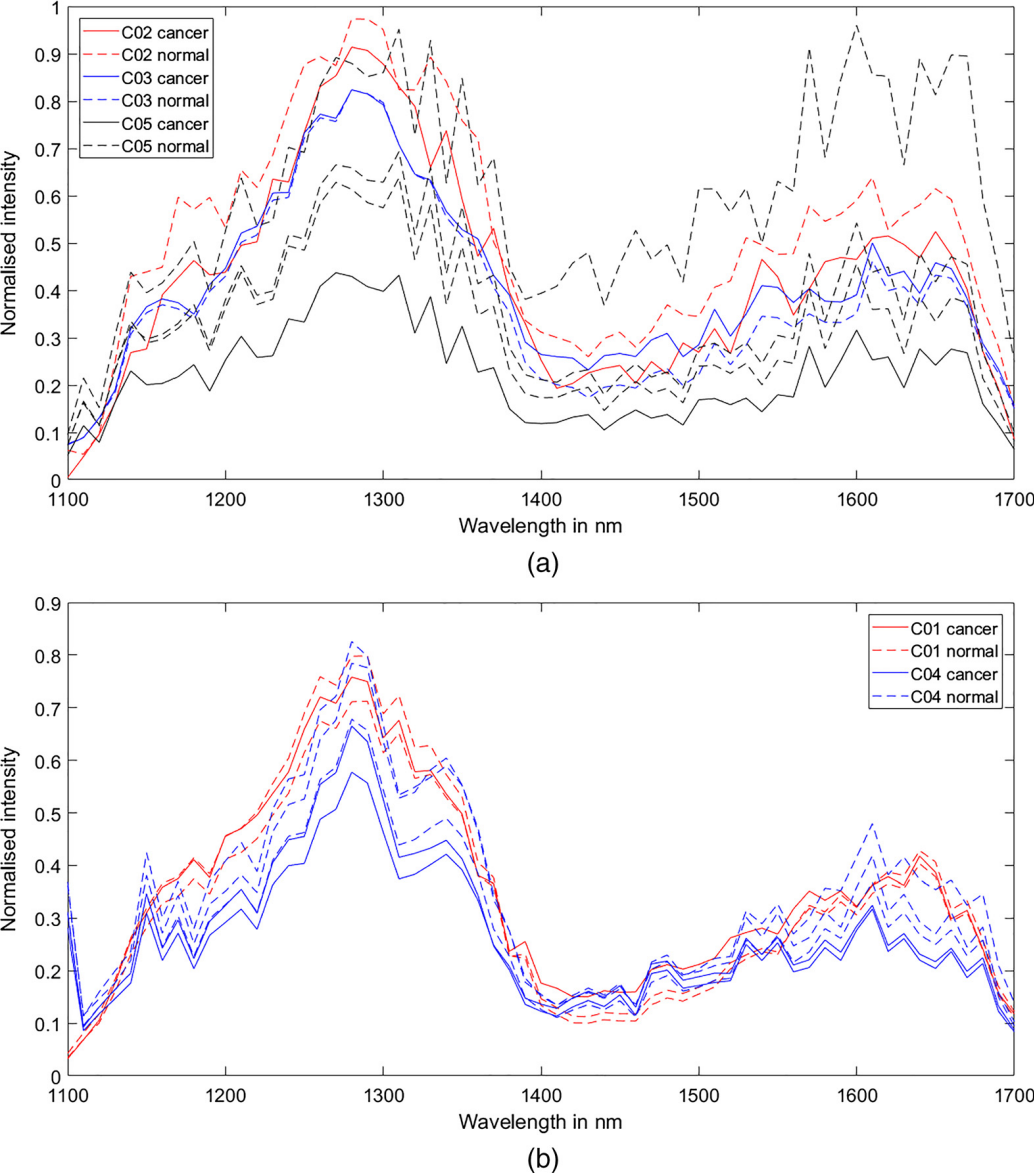
(a) ROI-derived absorption spectra of normal versus CRLM tissue obtained from three patients. The normalized values are based on single pixel maximum intensity. Therefore, the average values from ROI’s that are based on whole tissue specimen do not reach the maximum of 1 at any point. (b) ROI-derived absorption spectra of normal versus HCC tissue obtained from two patients.

Multispectral imaging was carried out on tissue samples from patients with CRLM (n=3) and HCC (n=2). Absorption plots were processed from ROIs that covered the whole area of an individual tissue sample. These were subsequently compared between normal and cancerous tissues [Fig. 5(a)]. As described in Sec. 2, the number of samples was determined by the size and nature of the liver resection specimen. At the conclusion of experiments, the tissue biopsies underwent histological evaluation, which was used to confirm the nature of the tissue sample (i.e., normal versus cancer). Visual analysis of the absorption curves did not reveal any obvious differences between cancer and normal tissues within the same patient although the samples from different patients did exhibit different characteristics. Mild liver steatosis was present in tissue from one HCC patient (C01). The absorption curve from this tissue exhibited a marked absorption peak, resulting in decreased pixel intensity, at around 1210 nm [Fig. 5(b)].

**Fig. 6:**
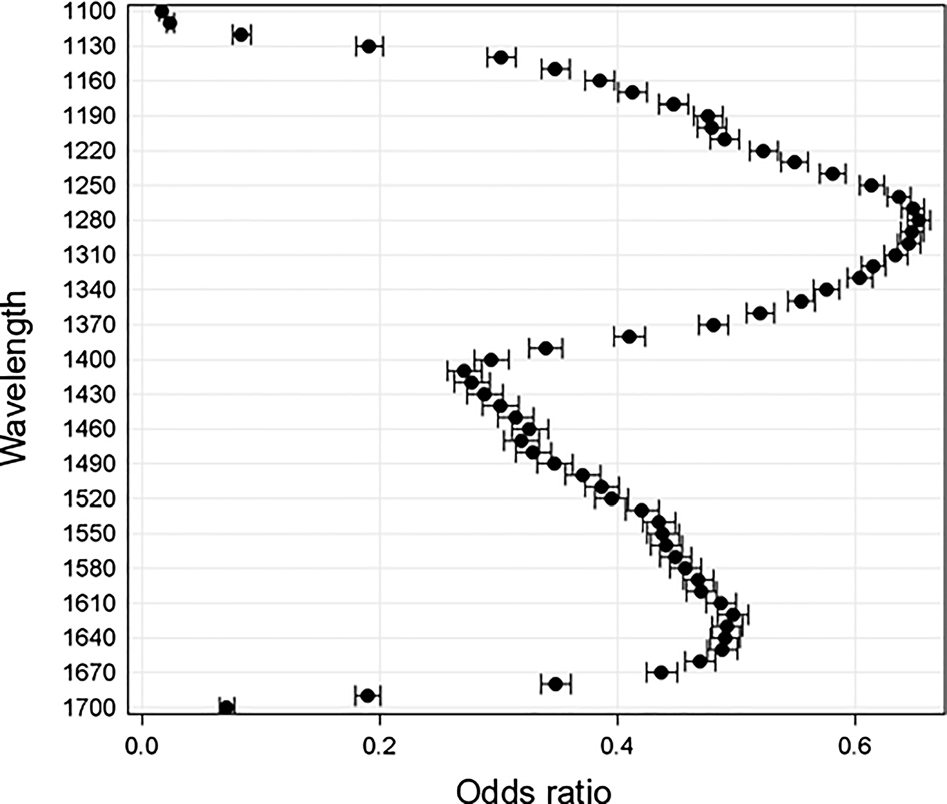
OR and 95% CIs for the effects estimates at each wavelength based on MTM analysis of CRLM tissue. OR changes are modeled on the assumption that normalized pixel intensity decreases from 0.66 to 0.41 (i.e., 25% increase in normalized tissue absorption).

A total of eight individual tissue specimen from three CRLM patients were analyzed (normal tissue n=5, cancer tissue n=3). This resulted in a total of 505.141 pixels being available for modeling. GLIMMIX analysis indicated that higher pixel intensities (i.e., a reduction in tissue absorption) may be associated with benign liver tissue. In other words, reduced tissue absorption made the presence of cancer tissue less likely. As opposed to results in HCC shown below, this held true for all measured wavelengths although the effect was more pronounced around 1400 nm, which is reflected by the OR estimates shown below (Fig. 6). For example, given a hypothetical increase of normalized pixel intensity from 0.41 to 0.66, the odds of cancer tissue being present at this pixel reduced by a factor of 0.39 (CI 0.37 to 0.4, p<0.001) at 1160 nm, whereas it reduces the odds by a factor of 0.65 (CI 0.64 to 0.66, p<0.001) at 1270 nm.

The impact of treating individual patients as a random effect did significantly contribute to the fit of the model with a covariance parameter of 0.6±0.51 (SE, P<0.001). The covariance parameter estimation indicates to what degree the random variable affects the fit of the statistical model (i.e., a higher estimate indicates a better fit) and its associated standard error gives a measure of repeat sampling variability.

#### 3.2.1 HCC

**Fig. 7:**
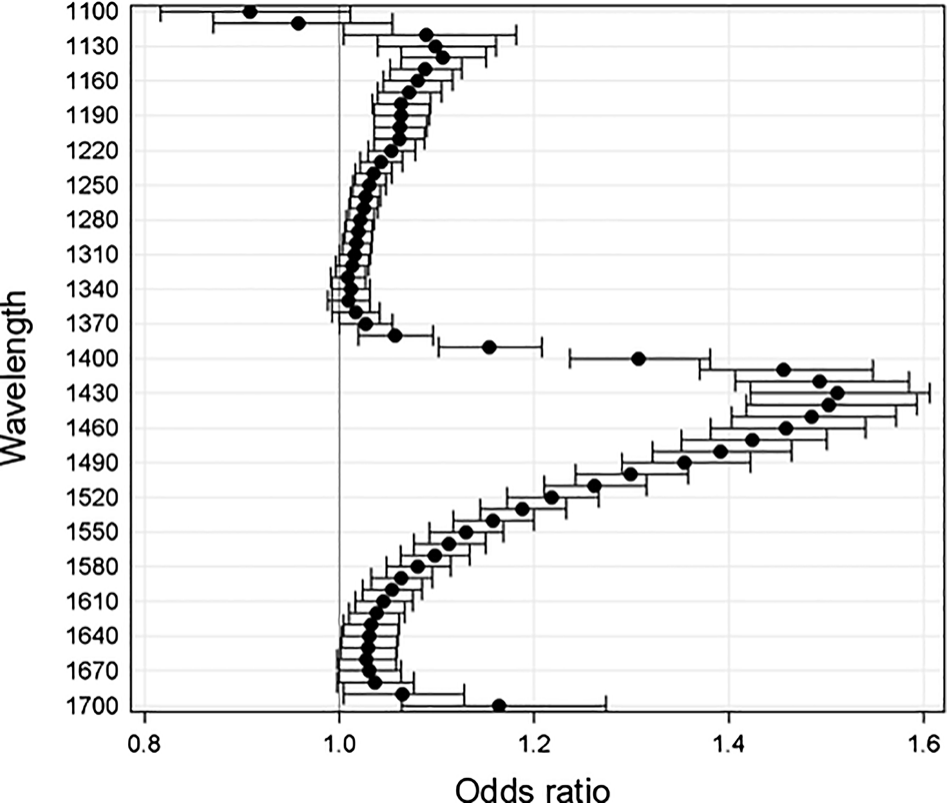
OR and 95% CIs for the effects estimates at each wavelength based on MTM analysis of HCC tissue OR changes are modeled on the assumption that normalized pixel intensity decreases from 0.57 to 0.32.

A total of eight individual tissue specimens from two HCC patients were analyzed (normal tissue n=5, cancer tissue n=3). This resulted in a total of 674.477 pixels being available for modeling. GLIMMIX analysis results contrasted those from CRLM tissue. In HCC tissue, higher pixel intensities (i.e., reduced absorption) are potentially associated with the presence of cancer tissue. In other words, reduced tissue absorption made the presence of cancer tissue more likely. As opposed to results from CRLM tissue, not all wavelengths between 1100 and 1700 nm were found to be associated with a tissue type (Fig. 7). The inclusion of patient ID as a random variable to the model did not significantly improve the fit of the model with a covariance parameter estimate of 0.154±0.154 (SE, p>0.1).

### 3.3 False Color Representation

Wavelengths where a strong tendency between absorption behavior and tissue type were indicated on GLIMMIX analysis and were selected for false color processing. Initial tests showed the resulting images to be very bright and lacking in features. To enhance the visual cues highlighting the tumor margins, additional wavelengths that exhibited a weaker association with tissue quality were included. It was hypothesized that normal and malignant tissues at these wavelengths would appear similar and hence would provide adequate contrast with wavelengths where the absorption in tumor and normal tissues was distinctly different. As far as possible, the wavelength was evenly spread throughout the recorded spectrum to better reflect the whole spectral range.

**Fig. 8:**
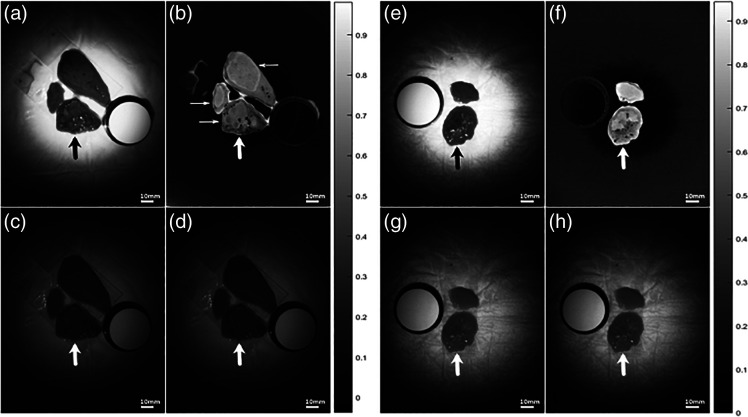
(a)–(d) Visualization of CRLM specimen (C03) including a representative color bar for all four images. The cancer specimen is marked with an arrow. (a) False color image based on mean intensity (1100 to 1700 nm). (b) False color image based on strong and weak predictive wavelengths. The thin arrow indicates a processing enhanced artifact effect that is created by the pressure of the microscopic glass slips on the tissue. Cancer tissue is indicated by the heterogeneous pattern on the center of the lower specimen. (c) Single wavelength image at 1280 nm. (d) Single wavelength image at 1420 nm. (e)–(h) Visualization of HCC specimen (C01) including representative color bar for all four images. The cancer specimen is marked with a wide arrow. (e) False color image based on mean intensity (1100 to 1700 nm). (f) False color image based on strong and weak predictive wavelengths. (g) Single wavelength image at 1280 nm. (h) Single wavelength image at 1420 nm.

Selected strongly associated wavelengths were 1130, 1410, and 1690 nm and 1140, 1400, and 1450 nm for CRLM and HCC specimens, respectively. Weakly associated wavelengths were 1180, 1340, and 1620 nm and 1250, 1370, and 1680 nm for CRLM and HCC, respectively. Pixel intensities in both association categories underwent numerical trapezoidal integration, which resulted in two separate intensity values for each pixel. Subsequently, the weakly associated intensity value was divided by the strongly associated intensity value to formulate a false color value. Other arithmetic methods (e.g., subtraction) were also evaluated but these yielded no contrast improvements. False color images were compared to single spectrum images selected at wavelengths of maximal (1420 nm) and minimal (1280 nm) absorption (Fig. 8).

Empirically, the false color images appeared to improve contrast between cancer and normal tissues. The gray level or color of a whole tissue specimen may occasionally appear quite similar between normal and cancer tissues, but based on the images in our study, the pixel intensities in cancer specimen appeared to be much more heterogeneous. In one patient (C05), no appreciable difference between normal and cancer tissues was seen in false color or single wavelength images.

## 4 Discussion

In this article, an image-based method of optical near-infrared spectroscopy for the evaluation of liver tissue has been presented. Utilization of a tunable light excitation source allowed cover of a broad continuous wavelength spectrum at intervals of 10 nm. Aspects include the systems capability to elicit pixel-based differences in absorption characteristics from organic tissues at submillimeter spatial resolution without requiring direct tissue contact. Because the tissue area represented by a pixel varies according to the imaging distance, the spatial resolution of MSI can be adjusted according to the desired amount of spatial detail.

Differences in absorption spectra may be obvious on visual inspection alone but this was not true for the comparison of malignant and normal liver tissues examined in this study. MTM data indicate a heterogeneous absorption pattern in malignant liver tissue that may enable it to be distinguished from benign tissue. To the best of our knowledge, this has not been reported in the literature before. When compared to benign tissue from the same patient, CRLM tissue exhibited increased heterogeneous absorption whereas decreased heterogeneous absorption was associated with HCC tissue. Aside from diagnostic applications, MTM may also have value as a prognostic tool. If absorption heterogeneity can be correlated with molecular markers of differentiation (e.g., Ki67, K-ras) in the future, then MTM could aid in predicting the biological behavior (i.e., growth and invasiveness) of cancer.

The prospect of identifying and mapping small or large areas of malignancy on the surface of the liver makes MTM a potentially attractive image guidance tool in oncological liver resection. As MTM is an image-based modality, it could be used in both open and laparoscopic surgery. Utilizing MTM to map the fat and collagen content of donor livers may be an attractive option for the assessment of donor liver quality prior to liver transplantation.^18^ False color visualization of liver tissue highlights the heterogeneous nature of liver cancer when compared to normal liver parenchyma.

In contrast to the work by Nachabe et al.,^1^ no attempt was made to estimate tissue concentrations of biological chromophores and to correlate these differences to the presence of pathology. Instead the method proposed here relies on the classification of tissues into malignant and normal followed by statistical analysis (GLIMMIX) to elucidate distinct absorption characteristics. This approach has the potential advantage of enabling the use of convoluted neural networks, to create exhaustive databases that contain the absorption signature of pathological and normal tissues. In this context, the automatic (i.e., user-independent) mapping of tissue pathology may become feasible. So rather than using observer-dependent tissue analysis based on chromophore concentration, absorption plots, or image color, an automatic recognition algorithm formulated from a database of stored tissue absorption signatures, could be implemented. In addition to tissue discrimination, such a database may also enable patient-specific prediction about the biological aggressiveness of cancer and hence determine the appropriateness of radical procedures. Although the system was only evaluated on liver specimen, it may also prove useful for analyzing tissues in other organs.^7^^,^^19^

A further advantage of MTM lies in the fact that it does not require direct tissue contact as is the case for the probe-based optical spectroscopy.^4^ In analogy to single spectrum-^20^ or filter-based multiband systems that have been evaluated by other groups,^8^^,^^9^ a tissue can be sensed at a distance. The advantage over these systems is that a broader wavelength range can be covered, which may in the future translate to improved discriminatory qualities. To the best of our knowledge, MSI of liver tissue with a spectrum range >450  nm has not previously been reported in the literature.

The results presented in this study have a number of limitations. First and foremost no attempt was made to prove a statistically significant relationship between absorption behavior and tissue type. Since the study was designed as a proof of concept for an imaging technique, only a limited number of samples were used, which meant that statistical validation was not feasible. It was felt inclusion of other liver pathologies would have made it difficult to gain an understanding on the capabilities of MTM and the best way of analyzing results. Another limitation is that the wavelength range examined from 1100 to 1700 nm did not include a number of biologically important absorption maxima related to the presence of hemoglobin or bile,^1^ which can be found in the range of 500 to 900 nm.^4^ Furthermore, a common limitation of optical imaging is a low penetration depth, which rarely exceeds 5 mm.^21^ This restricts the ability of MSI to evaluate deep lying tumors. It should, however, not impede the assessment of cancer resection margins as these are on the immediate surface. Also, it has been shown that removal of tumor within an area of <1  mm conveys a patient survival benefit in the resection of CRLM.^22^

Because the methodology employed in this paper can be applied to cameras and light excitation sources at shorter wavelengths, expanding MTM to wavelengths in the green and blue spectra should be technically possible. Detailed pixel-based analysis was mandatory because the absorption spectra of normal and cancer tissues did not exhibit readily appreciable differences. The data presented here suggest that further exploration of the discriminatory abilities of MTM on a large number of samples is warranted. Ideally, future studies should utilize a broader wavelength spectrum to incorporate absorption maxima of biologically relevant chromophores such as bile and hemoglobin. An increased information yield on the tissue composition of cancer may also allow correlation of absorption behavior with prognostic cancer variables, such as vascularity, differentiation, and treatment response. Because HCC and CRLM exhibited different absorption characteristics in this study, a future predictive algorithm would require *a priori* knowledge of the type of cancer, which is a limitation. In surgical practice, however, it is unusual to carry out liver resections without knowing the type of liver cancer.^23^^,^^24^

For logistical and ethical reasons, all experiments in this study were conducted *ex vivo*. It is important to test imaging concepts in a controlled environment first before expanding data collection to a more complex *in vivo* setting. This is especially true when involving high power laser sources that require specific precautions for eye protection as was the case in this study. The main difference anticipated in an *in vivo* setting will be the presence of oxygenated and deoxygenated hemoglobin, a difference that should not have affected our results because the spectrum from 1100 to 1700 nm does not include the absorption maxima for hemoglobin. Furthermore, it is important to consider that all published data on the optical spectroscopy of human cancer so far have been conducted *ex vivo*. Therefore, our results are more relatable to published data from other authors.^4^

The advantage of the MTM system is that it can be finely tuned to the desired wavelength enabling the acquisition of detailed absorption spectra. A current disadvantaged is the large bulk of the system and laser safety considerations. Therefore, an important step in the future research is to create a more mobile and perhaps miniaturized version of the system that can be utilized in the operating theater.

Pending further research, it may become possible to conduct MTM with a combination of several single spectrum instead of utilizing multiple spectra in continuity as in this study. This may allow the construction of MTM systems that employ optical filters in combination with broad spectrum white light sources or a combination of several single wavelength laser sources. Either option could translate to a more compact MTM system. A broad-spectrum tunable system as developed in this study may then act as a research platform to inform the construction of simpler systems for clinical use.

## 5 Conclusions

In summary, this paper has described the development of a concept for MSI of the liver called MTM. It was demonstrated that tiny areas represented by a single image pixel may exhibit differences in absorption characteristics, which may enable tissue discrimination at a submillimeter dimension. Important areas for future development include the *ex vivo* evaluation of specific disease entities and the *in vivo* evaluation of MTM.
